# 

**Published:** 1989-02

**Authors:** 


1 **Contents**
2 **Editorial**
3 **Malformation, mutation and malignancy**
Martin Mott
11 **A Bristol experience: Benefits and Cost of an "Asthma Nurse"**
F. Carsewell, E. J. Robinson, G. Hek and T. Shenton
13 **Susan Britton Wills Unit, Bristol General Hospital, multidisciplinary, general hospital and Community Psychiatry**
Glin Benet, David Cook, Jan Smith
15 **Are you being served? A survey of the opinions of G.Ps. referring patients to the Bristol Dental Hospital**
B. G. H. Levers, C. M. Scully and R. W. Matthews
16 **Pancreatic Transplantation, the 8th Marjorie Budd Lecture**
Peter Morris
17 **The Empoisoned Darts of Venus**
John R. Guy
19 **New Dimensions in the N.H.S.**
J. L. Burton
21 **From our Correspondents**
23 **Letter to the Editor**
24 **Surgical Club of S.W. England, Meeting at Gloucester and Cheltenham, November 1987**
27 **South West Orthopoedic Club, Meeting at Taunton, November 1987**
29 **South West Paediatric Club, Meeting in Bristol, October, 1987**
31 **West Country Chest Society, Meeting in Exeter, March 1988**

